# Climate change alters impacts of extreme climate events on a tropical perennial tree crop

**DOI:** 10.1038/s41598-022-22967-7

**Published:** 2022-11-16

**Authors:** Thomas J. Creedy, Rebecca A. Asare, Alexandra C. Morel, Mark Hirons, John Mason, Yadvinder Malhi, Constance L. McDermott, Emmanuel Opoku, Ken Norris

**Affiliations:** 1 Department of Life Sciences, Natural History Museum, London, UK; 2 Nature Conservation Research Centre, Accra, Ghana; 3 Department of Geography and Environmental Sciences, University of Dundee, Dundee, UK; 4 Environmental Change Institute, School of Geography and the Environment, University of Oxford, Oxford, UK; 5 Ghana Cocoa Board, Accra, Ghana

**Keywords:** Climate-change impacts, Agroecology

## Abstract

Anthropogenic climate change causes more frequent and intense fluctuations in the El Niño Southern Oscillation (ENSO). Understanding the effects of ENSO on agricultural systems is crucial for predicting and ameliorating impacts on lives and livelihoods, particularly in perennial tree crops, which may show both instantaneous and delayed responses. Using cocoa production in Ghana as a model system, we analyse the impact of ENSO on annual production and climate over the last 70 years. We report that in recent decades, El Niño years experience reductions in cocoa production followed by several years of increased production, and that this pattern has significantly shifted compared with prior to the 1980s. ENSO phase appears to affect the climate in Ghana, and over the same time period, we see corresponding significant shifts in the climatic conditions resulting from ENSO extremes, with increasing temperature and water stress. We attribute these changes to anthropogenic climate change, and our results illustrate the big data analyses necessary to improve understanding of perennial crop responses to climate change in general, and climate extremes in particular.

## Introduction

Changes in the patterns of climate and climate extremes through anthropogenic climate change will cause substantial changes to crop production^1^, and understanding the processes that shape these responses is increasingly important to maintain food supplies and the livelihoods that depend on farming, distribution and industrial processing of crops. This is particularly true in the global south where a greater proportion of farmers live at or below the poverty level and there may be less state, institutional and individual resilience to production volatility. Worse, the relatively stable intra-annual climate of the tropics is most at risk of experiencing novel climatic conditions as a result of climate change^2^. These conditions may first be experienced as a result of climatic oscillations such as those driven by the El Niño Southern Oscillation (ENSO), which is increasing in frequency and magnitude^3,4^. Understanding the links between ENSO and crop production may contribute to the monitoring and prediction of crop production, informing management of agriculture and markets, and potentially providing early warnings for disruption to livelihoods from widespread crop failures.

El Niño events bring hot weather to much of the terrestrial tropics, often accompanied by reduced rainfall^5^; the resulting droughts reduce vegetative productivity and have increased in severity under climate warming^2^. The impact of ENSO phase on crop production has been demonstrated at spatial resolutions from small-scale farm studies (e.g. rice^6^, coffee^7^, cocoa^8^) disentangling vegetative responses to management, pests, disease and climate, to regional and national production^9,10^ exploring the substantial geographic variation within responses at regional and global scales^11^. Much crop-ENSO research has focused on annuals, the source of the majority of the world’s food, and the short life cycle of these crops allows for direct inference of the impact of climate shocks. Perennial crops, particularly tree crops, have received less attention, despite the US$538tn 2019 gross production value of perennial tree crop agriculture globally^12^ and the importance of these crops to livelihoods^13^. The possibility of delayed impacts of ENSO over the multi-annual life cycle of perennial crops further highlights the need to address this research gap.

**Figure 1:**
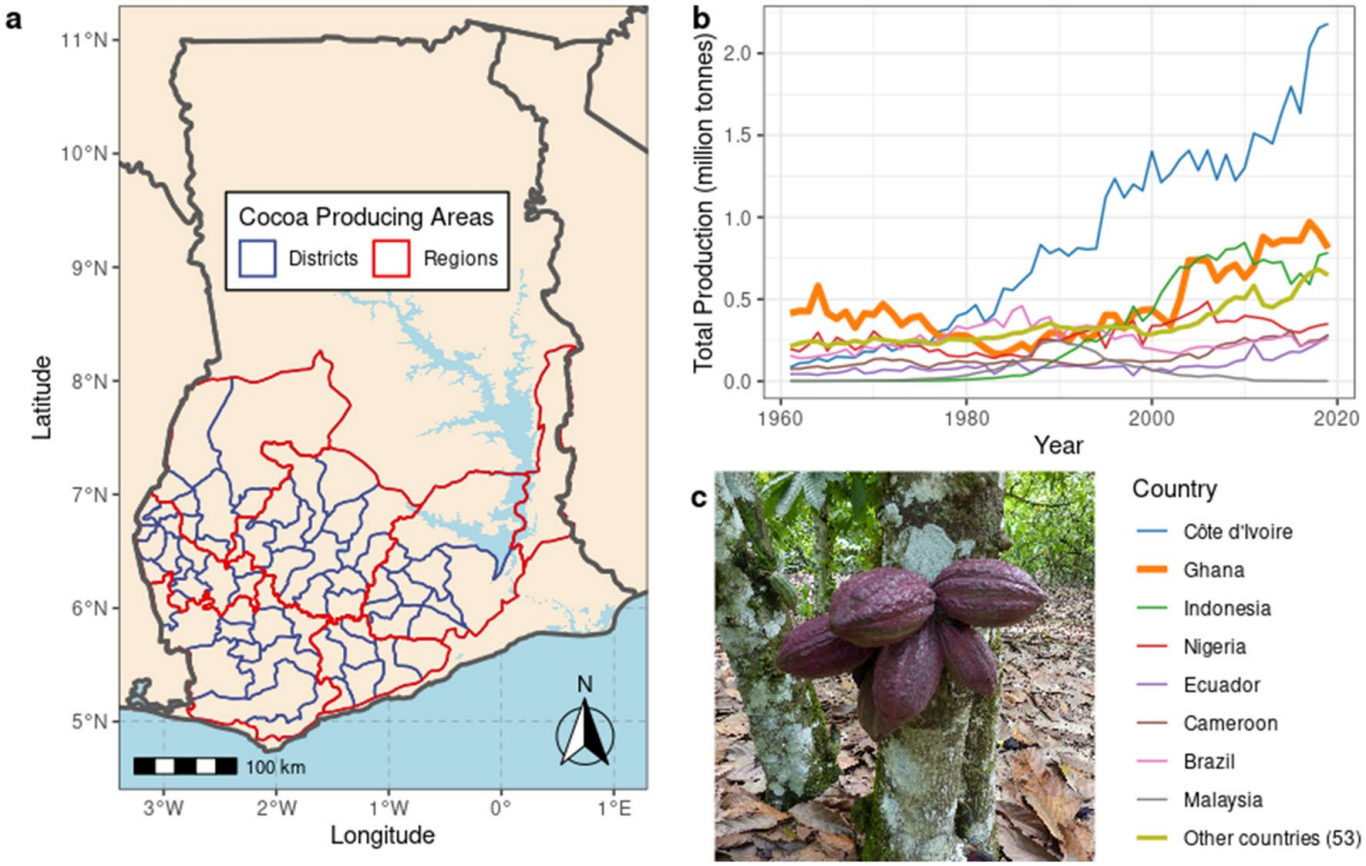
Cocoa production in Ghana. (**a**) Map of cocoa producing districts (blue outlines) and regions (red outlines) in Ghana. (**b**) Global cocoa production over the last 60 years. (**c**) Cocoa pods on a farm in Ghana (photo copyright A.C.M).

Here, we use a novel big data approach for understanding the impact of ENSO phase on perennial tree crops using long term data of a model system: cocoa agriculture in Ghana. Cocoa (*Theobroma cacao* L., Fig. 1a–c) is grown throughout the tropics by 5–6 million farmers, with 90–95% of production from smallholder farms of 3 hectares or less^14^. Ghana and neighbouring Cote d’Ivoire, sharing a similar climate and ecology, are the world’s top cocoa producers^12^ (Fig. 1b) in a global raw market worth US$8.2bn in 2019. ENSO phase has been observed to impact West African climate^5,15^ and reported to impact cocoa production^16^, although no climatic teleconnection has yet been discovered^17^. As the raw material of a major global food industry, the implications of volatility in cocoa production reach beyond farmers to affect major cocoa-producing states and multinational companies.

There is hence a clear knowledge gap in our understanding of ENSO and climate change impacts on local climate in West Africa and perennial tree crop production. In this paper, we investigate (1) the instantaneous and delayed responses of cocoa production to ENSO phase, (2) change in these responses over time and (3) the local climatic impacts of ENSO phase to identify potential climatic drivers of cocoa production during climate shocks.

## Materials and methods

### Cocoa production data acquisition

Cocoa production data was furnished by the Ghana Cocoa Board, comprising the annual total weight of cocoa beans purchased in the 6 cocoa purchase regions for every purchase year from 1947/48 to 2019/20 (excluding 1976/1977 for two regions, Supplementary Table 1), and in the 68 cocoa purchase districts for every purchase year 1999/2000 to 2019/2020 (Fig. 1a). Purchase years run from late September/early October; we assumed the purchase year ran from 1st October to 30th September. Production weights varied substantially between administrative divisions, and within administrative divisions over time, presumably in the most part due to variation in the area under production (AUP), for which no data was available. To control for the effect of varying AUP, and other effects such as technological improvements in farming practice, the production data was detrended by conversion to z-scores, i.e. the number of standard deviations from the mean or expected value. For district data, z-score calculation was performed based on a linear best fit line for each district, i.e. the z-score for a particular observation was the number of standard deviations from the expected value for that year derived from the slope; for regional data a linear relationship was not appropriate so z-score calculation was performed based on a 9-year rolling average.

### ENSO data acquisition

To identify ENSO phase, particularly extreme El Niño and La Niña events, we acquired the complete Oceanic Nino Index (ONI) dataset NOAA^18^, comprising rolling 3-month running means of SST anomalies in the Nino 3.4 region from 1950 to 2020. This data was summarised for each purchase year (Oct–Sep, see above) by taking the value of the greatest magnitude (retaining the sign) within each purchase year, referred to as maximum annual magnitude of ONI (mamONI).

### Climate data acquisition

We acquired ERA5 climate data^19^ on temperature, precipitation and evaporation at 0.25° resolution from 1950 to 2020. From these data we computed monthly and seasonal mean, minimum and maximum temperatures, total precipitation and Cumulative Water Deficit (CWD)^5,20^ for each grid cell (Supplementary methods). We defined Ghana’s four climatological seasons as: minor wet—September and October; major dry—November to March; major wet—April to July; minor dry—August. The minor wet season crosses the purchase year: we considered this as falling at the beginning of the purchase year rather than the end, as this has a more reasonable link to cocoa production. Finally, each climate metric was converted to anomalies by subtracting the mean value for the metric for a reference period, set to 1981–2010 to encompass only data from the final release ERA5 dataset.

The monthly and seasonal summary raster bricks were filtered to include only cells that intersected with Ghana’s cocoa growing areas and comprised less than 15% permanent water bodies, based on the observation that cells including the Atlantic ocean or the Volta river/reservoir formed substantial outliers for some climatic variables. Filtering used spatial polygons of the Ghana cocoa regions supplied by the Ghana Cocoa Board, the Ghana coastline^21^, and Ghana water bodies^22^.

All GIS data manipulation and computation was performed in R 4.0.5^23^ using the sf^24^ and stars^25^ geospatial packages and their dependencies.

### Cocoa production analysis

All analysis was performed in R 4.0.5^23^. To identify possible delays in the relationship between ONI and production we computed, separately for each district or region, the cross-correlation of the production anomaly time series against the mamONI time series for delays of 0–12 (i.e. production anomaly against mamONI values for the current and 12 preceding years) and computed the probability of each correlation coefficient differing from 0. For each dataset (district, regional), we then calculated the mean of all correlation coefficients for each of the 13 delays, and computed student’s t to test if this mean was significantly different from 0. To ensure that the detrending methodology had sufficiently standardised the production data over time for regression to be appropriate, we conducted, separately for each district or region, a search of ARIMA models to ensure that the best fitting parameters for the order of autoregression, degree of differencing and moving average were all equal to 0. This search was implemented in the auto.arima function from the forecast R package^26^, fitting mamONI as an external regressor, using AIC to compare candidate models and using non-stepwise selection and no approximation of information criteria for intermediate models to improve accuracy. For both district and regional data, the majority of time series had parameter values of 0 for all three parameters (Supplementary Table 2). To ensure that the detrending methodology had sufficiently standardised the production data over space, i.e. by removing confounding inter-district or inter-regional variation in production, we checked the singularity of a mixed effects model for each dataset, fitting detrended production against the intercept with district or region as a random effect using the isSingular and lmer functions from the lme4 package^27^, expecting that if detrending sufficiently removed variation in the random effect, the resultant random effect variance would be close to zero and cause singularity.

To assess the contribution of different delayed mamONI values on the district-level production dataset, we performed multiple regression with production anomalies as the response variable and mamONI at delays of 0–3 years as additive explanatory variables. For the region dataset, we additionally wanted to explore the extent to which the response of production to variation in mamONI has changed over time. To this end, we generated 71 two-factor categorical variables that each grouped observations into before/after each year in the dataset, to examine inflection points in the response of production to ONI. We then created a set of candidate models as follows: (1) the same model as used in the district data, with four delays (0–3); (2) model (1), but with year (centred and scaled to unit variance) as an interaction with each delayed mamONI variable; (3) 71 piecewise regressions, each based on model (1) but with one of the year grouping variables as an interaction with each delayed mamONI variable. The 73 candidate models were compared using AICc, implemented in the model.sel function of the MuMIn R package^28^.

### Climate analysis

We analysed the processed ERA5 climate data to explore the climatological relationships between the Oceanic Nino Index and cocoa production through (1) identifying instantaneous and delayed climatic responses in the cocoa producing areas of Ghana to ONI-defined El Niño and La Niña events and (2) identifying any shifts in these responses over the same time period as identified by the cocoa production analysis. To identify possible delays in the relationship between ONI and climate we computed, separately for each climate metric, the cross-correlation of the climate metric anomaly time series against either monthly ONI values (for monthly climate metrics) or against mamONI values (for the purchase year corresponding to seasonal climate metrics). Cross-correlations were performed for leads/delays between − 36 and 36 months or − 3 to 3 years, as appropriate; statistical tests were then performed as described for the cocoa production data. We then regressed monthly and seasonal climate metrics against mamONI values separately within month and season, and examined these relationships for changes over time by fitting interactions with the year grouping variable identified in the cocoa production analysis.

## Results

**Figure 2:**
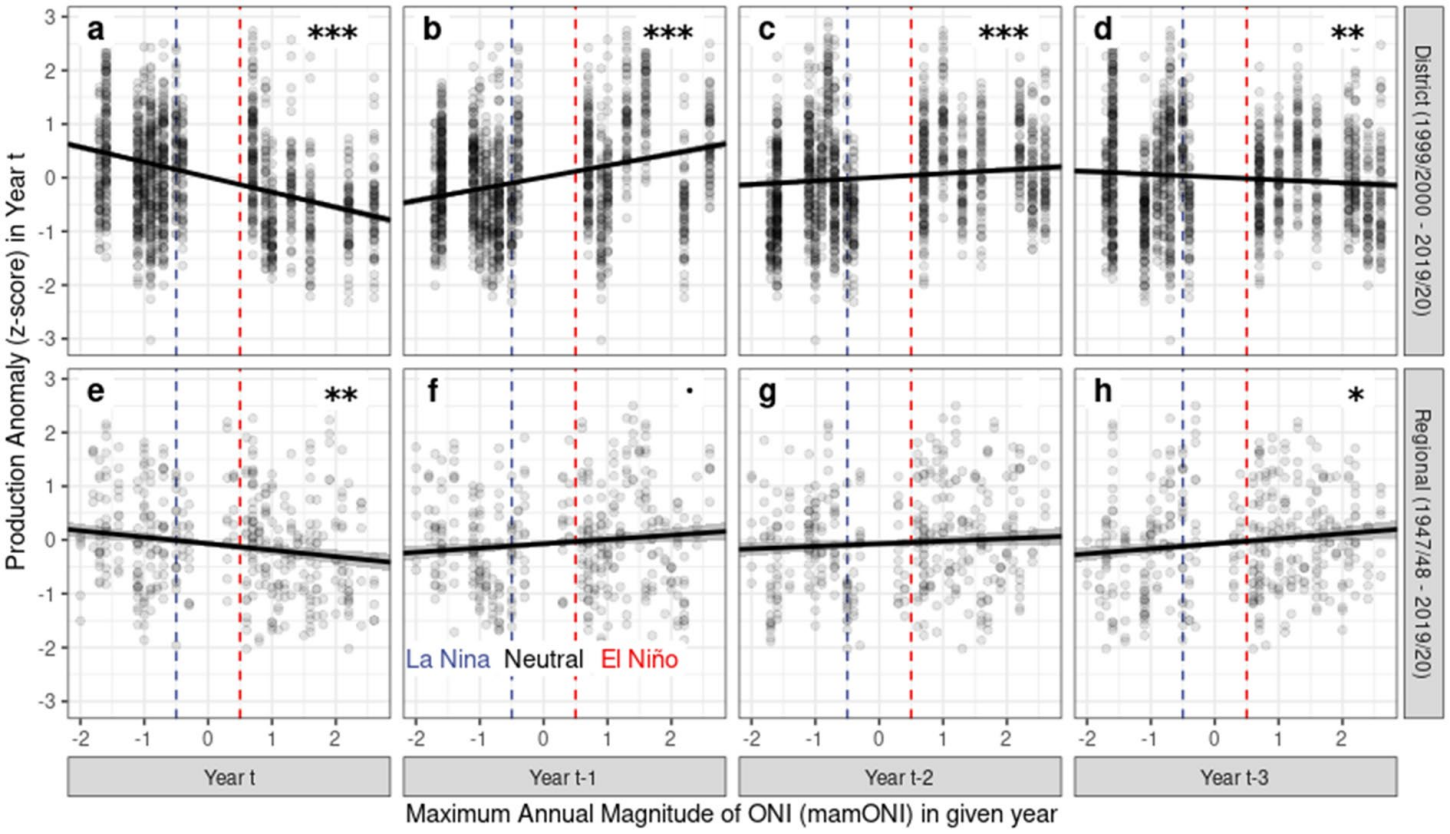
The instantaneous and delayed responses of cocoa production to ENSO phase, represented as mamONI. Vertical dashed lines delineate La Nina (mamONI ≤ − 0.5), Neutral (− 0.5 < mamONI < 0.5) and El Nino (mamONI ≥ 0.5) conditions. The relationships between Production Anomaly and mamONI through time are explored using a multiple regression for each dataset (panel rows: District dataset, 1999/2000–2019/2020, **a**–**d**; Regional dataset, 1947/1948–2019/2020, **e**–**h**) fitting the Production Anomaly in year t against mamONI in years t to t-3 (panel columns). Lines show the best linear fits and standard errors derived from each multiple regression. Significance stars denote the probability that a slope differs from zero (***p < 0.001, **p < 0.01, *p < 0.05, .: p < 0.1). Adjusted R squared for the District model was 0.20, for the Regional model 0.05.

We expected from previous smallholder surveys in Ghana^16^ to see declines in cocoa production in El Niño years. Using the highly replicated dataset of cocoa production over 68 districts for the past two decades, we fitted multiple regressions of detrended production in year *t* against mamONI for the same and 3 prior years (i.e. years *t* to *t − 3*). These showed that mean detrended production significantly declined with increasing mamONI in year *t*, i.e. production exceeds that expected by the district-wise trend during La Niña years and is lower than the average trend during El Niño years (Fig. 2a). We also see significant relationships between mean detrended production and mamONI in years *t − 1*, *t − 2*, and *t − 3* (Fig. 2b–d)*,* indicating delayed effects on production, with increased mean production anomaly in the two years following an El Niño year.

**Figure 3:**
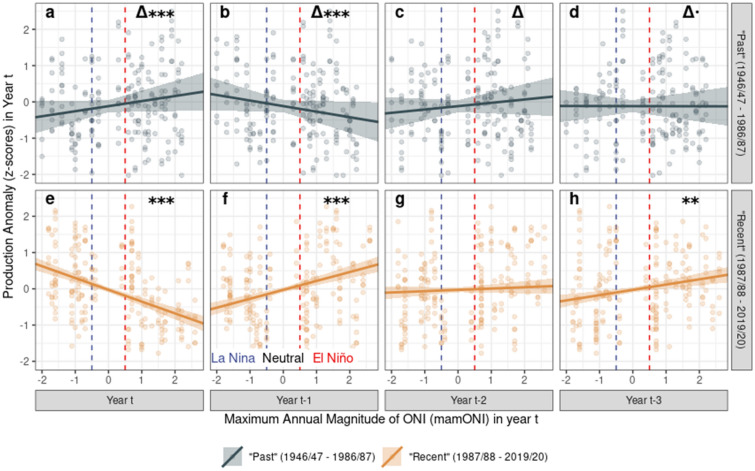
The changing response of cocoa production to mamONI over time in the Regional dataset, separated into “past” and “recent” purchase years. Vertical dashed lines delineate La Nina (mamONI ≤ − 0.5), Neutral (− 0.5 < mamONI < 0.5) and El Nino (mamONI ≥ 0.5) conditions. Lines show the best linear fits and standard errors derived from a single ANCOVA model fitting a two-level factor splitting the data into two time periods as an interaction with each of the four year delays. Significance stars denote the probability that: top row (**a**–**d**)—the slopes for each year (columns) differ from one another; bottom row (**e**–**h**)—the slope differs from zero (both rows—***p < 0.001, **p < 0.01, *p < 0.05, .: p < 0.1). Adjusted R squared for the ANCOVA model was 0.17.

Fitting the equivalent models to the regional data for the past 70 years, we report a similar pattern to the district analysis, but the significant instantaneous negative effect is reduced in magnitude (Fig. 2e), and all terms are less significant (Fig. 2f–h). This difference arises because the production response to ENSO has changed over this time period: comparison of candidate models allowing the response to mamONI to vary in time returned a best model that fitted a break-point in the ENSO-production relationship between the 1986/1987 and 1987/1988 purchase years, with other high-scoring models fitting break-points between 1985 and 1988 (Supplementary Table 3). Hence, since the mid-1980s (“recent”), the ENSO-production relationship mirrors that of the district data (Fig. 3c–h), but prior to this (“past”), patterns of production in relation to ENSO were significantly different (Fig. 3a–c). Namely, prior to the 1980s, El Niño years brought production exceeding that expected by the district-wise trend, and vice versa during La Niña years.

**Figure 4:**
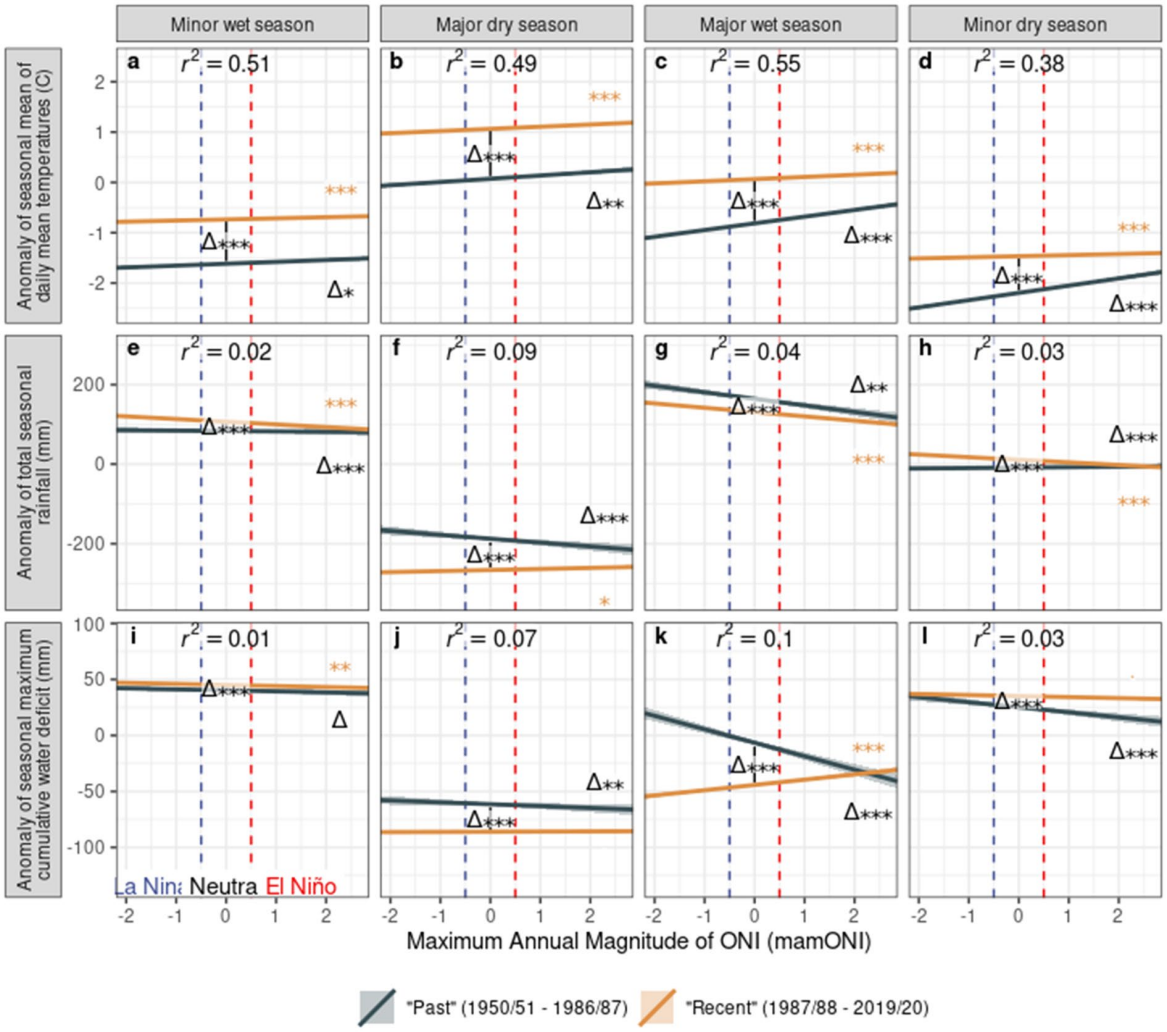
The response of climate to mamONI in different seasons during the purchase year, grouped into two sets of years corresponding to the best fitting break-point in the production data. Seasons are shown in chronological order during the purchase year. Vertical dashed lines delineate La Nina (mamONI ≤ − 0.5), Neutral (− 0.5 < mamONI < 0.5) and El Nino (mamONI ≥ 0.5) conditions. Lines show the best linear fits and standard errors derived from 12 individual regressions of seasonal climate against mamONI, with an interaction term fitting the year category. Significance stars denote p-values derived from these models (***p < 0.001, **p < 0.01, *p < 0.05, .: p < 0.1): (1) difference in means between year groups (delta in centre of plot), (2) difference of the 1987/1988–2018/2019 slope from 0 (orange stars at right of plot), (3) difference between slopes (delta at right of plot). The adjusted R squared value is displayed for each model.

The impact of ENSO on cocoa production is mediated through climate, thus we sought to examine the ENSO-climate relationship in Ghana’s cocoa production zone during the purchase year, and explore the extent to which this relationship may also have changed over the time period of our long-term production dataset. The regression results show that in “recent” purchase years (orange lines, Fig. 4), El Niño conditions cause significant increases in temperature across all seasons and decreasing rainfall in most seasons, particularly the major wet season; conversely, La Niña conditions bring cooler, wetter conditions. Drought stress responds accordingly: El Niño brings significant increases in drought stress (lower MCWD) compared with La Niña in most seasons, although the effect is slight.

Comparing “past” and “recent” climatic responses to ENSO phase (blue vs orange lines, Fig. 4) shows significant increases in mean temperature throughout the year, so while the magnitude of the warming trend has either not changed or lessened, “recent” El Niños nonetheless bring mean temperatures not experienced in the “past”. Rainfall has changed less substantially over time; while the changes in mean rainfall are significant, they remain small, apart from in the major dry season which has become substantially drier over time. This results in a significant decrease in mean MCWD in the major dry season between “past” and “recent” years, denoting greater drought stress (Fig. 4j). In general, across all metrics and seasons, the slopes of the effect of mamONI on climate metrics are shallower in “recent” years compared with the “past”, suggesting that ENSO phase now drives less climatic variation among ENSO phases (between El Niño and La Niña years) than in the past.

**Figure 5:**
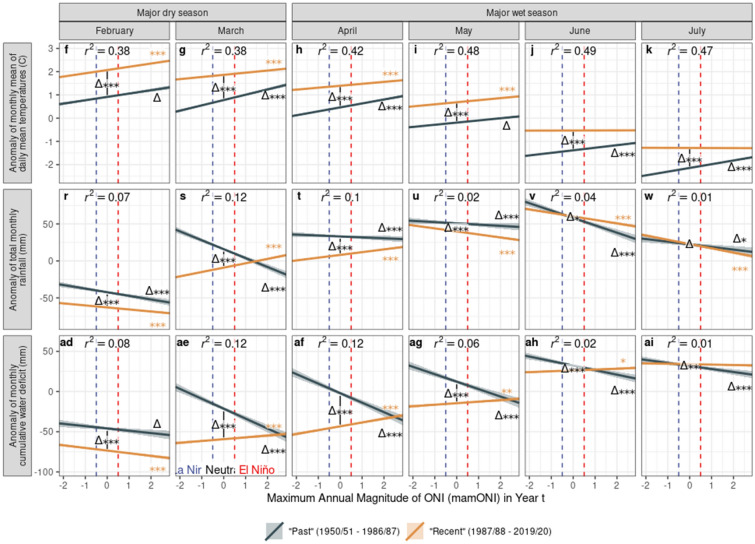
The response of climate to mamONI in selected months during the purchase year, grouped into two sets of years corresponding to the best fitting break-point in the production data. Supplementary Fig. 1 shows a complete version with all months; panel letters are consistent across this plot and Supplementary Fig. 1, hence the missing letters in this plot. Lines show the best linear fits and standard errors derived from individual regressions of monthly climate against mamONI, with an interaction term fitting the year category. All colours and notations as in Fig. 4.

MCWD has significantly reversed direction during the major wet season between “past” and “recent” years (Fig. 4k). In the “past”, El Niño brought increased drought stress, as expected by the warmer, drier conditions (Fig. 4c,g), while in “recent” years drought stress appears to *decrease* during El Niño, despite the same conditions. This result appears counterintuitive; however the monthly analyses (Fig. 5, Supplementary Fig. 1) show an ongoing impact on CWD of significant changes in rainfall earlier in the year, namely a reversal in direction of the rainfall response to ENSO phase during March and April (Fig. 5s,t). El Niño brings increased rainfall in “recent” years compared with decreased rainfall in the “past”, reflected in the March and April CWD (Fig. 5ae,af), and this increase, coupled with generally increasing average rainfall and slightly decreasing average temperature entering the major wet season, results in decreased CWD for several months.

## Discussion

Using a robust recent dataset, our analyses show that cocoa production is significantly affected by the maximum magnitude of ENSO phase during the current and previous purchase years (Fig. 2). The instantaneous effect is negative, followed by delayed positive effects in the two following years and negative in the third following year, combining to give a picture of multi-year fluctuations in cocoa production as a result of El Niño/La Niña events. Using a 70-year dataset, we show significant changes in these instantaneous and delayed ENSO-production relationships between recent and past time periods (Fig. 3). Using ERA5 data for the cocoa production area of Ghana, summarised at the same temporal resolution as the production data, we demonstrate significant relationships between ENSO phase and climate, with significant changes in mean climate and in ENSO-climate relationships (Fig. 4) between recent and past time periods. This agrees with prior work suggesting that ENSO may impact West Africa^5,15^, despite no current evidence of teleconnections between ENSO phase and West African climate^17^.

Our 70-year production dataset represents a temporal extent unmatched by other research, however was aggregated to fewer replicates than the 21-year analysis (6 regions vs 68 districts). While this may represent reduced power, results from the overlapping time period of the two datasets strongly agree. The computation of yield, a more comparable metric between different-sized areas than total production, was not possible because data on area under production (AUP) were not available. However, the detrending process employed successfully eliminated variation between districts or regions (of which AUP is likely a substantial component) and long-term technological trends that would otherwise confound our ability to isolate the ENSO signal (Supplementary results).

Perennial crops have multi-year growing patterns, with allocation of resources to growth, development and reproduction driven by climate in ways that are not fully understood^29^. ENSO generally peaks between October and December, also the busiest cocoa purchase period: thus we observe a relatively instantaneous apparent effect of ENSO phase on cocoa production. This reduction in cocoa production under El Niño inis consistent with results from farm monitoring^8^ and large-scale farm surveys^30^ evidencing production declines in from other regions (where teleconnections are understood), and with analyses of production data from West Africa^31^. During the main cocoa purchase period, coinciding with the minor wet and major dry seasons, we observe increases in water deficit during El Niño, leading to drought stress conditions. In small-scale cocoa studies, drought stress is correlated with reduction in pod production and increased tree mortality^8,32^, and in similar studies of other tree crops drought is directly linked to reduction in fruit or nut production^33^, although in all cases the mechanisms are unclear. Drought may generally create unfavourable conditions for growth and reproduction through reduced availability of water for vital processes, or more specifically by promoting disease incidence and pod rot^8^, increasing the chance of fire, increasing competition for soil moisture^32^, and/or reducing pollinator populations^34^. Alternatively, cocoa may respond to reduced water availability by reallocation of resources away from energetically expensive reproduction: rainfall exclusion experiments suggest that in the medium term, while bean production drops, vegetative growth is not significantly reduced during drought^32^.

The significant increases in mean temperature and average drought stress we observed in some seasons over time is such that the climate experienced during El Niño events in recent decades represent novel extreme conditions for Ghana’s cocoa agriculture. This causes significant changes in the responses of cocoa production to ENSO phase over the same time period. One explanation for this may be that the warm, dry El Niño conditions in Ghana in the past were within the environmental tolerance of cocoa, leading to allocation of resources to reproduction in response to drought, increasing cocoa bean production and resulting in less severe instantaneous and delayed responses to ENSO phase (Fig. 3a–d) However, in recent decades this level or greater drought stress has become the norm (Fig. 4i–l), with El Niño conditions apparently triggering a different response mode, allocating resources away from reproduction in the short term and creating oscillating resource allocation over the following years.

However, understanding the delayed responses of cocoa is challenging, especially as these represent a novel finding. There is little research that explores multi-annual physiological or ecological responses of cocoa to drought, and the explanation is likely to be a combination of both residual/delayed climatic responses to ENSO phase, and of life history strategies. The observed increase in production during the two years following El Niño may be explained by post-drought reallocation of resources to reproduction as remediation for lost reproductive output in the instantaneous response, or a shift to a ‘faster’ strategy by allocating resources to reproduction over the longer term, becoming evident in the data in subsequent years. Alternatively, this may be explained by favourable climatic conditions occurring during an El Niño event that impact the following years’ crop. March and April is a crucial time for cocoa pod development in Ghana and in recent years El Niño appears to bring greater rainfall during these months. Given the 6–9 months development of cocoa beans, the effects of this increased rainfall and reduced water deficit on cocoa production will be seen in the delayed response. We see evidence of this in the climate-change driven reversal of March–April rainfall patterns: while in the past El Niño has consistently resulted in drought stress, this reversal provides a respite from drought, buffering trees from reduced rainfall during the major wet season and giving sufficient resources for improved production in the following year.

The robustness of our results provide evidence that may aid development of resilience strategies for ENSO-driven cocoa production variation in Ghana, but we may also consider whether these results can be generalised to the production of cocoa and/or perennial tree crops globally. The climatic impact of ENSO observed in Ghana is broadly consistent with many regions of the tropics^2^, the instantaneous cocoa production responses to El Niño are consistent with findings in these regions, and so we may expect these regions to see a similar pattern of multi-annual cocoa production variation in response to ENSO phase. However, there is considerable variation in ENSO responses among and within other perennial tree crops in regions where climatic responses to ENSO are similar to Ghana. Oil palm yields have been negatively associated with ENSO phase in Malaysia^9^, as have olive yields in Morocco (delayed by a year)^33^. Conversely, apple yields have been positively associated with ENSO phase in China^10^, as have coffee yields in Brazil^35^; however, no effect at all is seen in coffee in India over a 35-year time series^7^. Most of these analyses considered only a single ENSO phase (usually El Niño), and most considered only instantaneous impacts. However, it is clear that most of these crops do respond to ENSO, and given the shared biology it is reasonable to assume that delayed effects of ENSO phase are likely and should be considered to understand the full picture of ENSO impacts on perennial tree crops.

The larger body of research into ENSO impacts on annual crops includes many studies using long time series, reporting high heterogeneity in space and among crops^11,36,37^. However, there appears to be little examination of changes in the direction and magnitude of ENSO responses over time; thus our findings are timely and signal that further research is needed to examine how changing climates may force novel extreme climatic conditions and shift response patterns to ENSO phase. Given that perennial tree crops are generally cash crops, and the utility of these crops to farmers are to a greater or lesser extent mediated by market forces, there is a need for improved forecasting of yield in response to changing climate and ENSO patterns to withstand production fluctuations. The low perishability of many perennial tree crops means that with accurate forecasting, supply may be managed or even exploited to ensure consistency of income both for farmers and those whose livelihoods depend on related food manufacturing industries.

Our approach to understanding the responses of a perennial tree crop to ENSO phase and anthropogenic climate change exploited existing global, national and subnational datasets for climate and production with appropriate spatial and temporal resolution. We use freely available geographic and climate data, and employ highly replicable methods: a simple pipeline of climate data aggregation and summary computation, coupled with standard detrending and straightforward analytical methods with a relatively small computational requirement. This “big data” approach to agriculture-climate research demonstrates a relatively straightforward framework for understanding responses of agricultural productivity to climate and identifying temporal changes in these relationships. While small-scale studies examine the mechanisms of climate impacts through the interacting effects of agricultural practices, abiotic conditions, disease incidence and multi-trophic interactions, large-scale studies across regions and over time scales encompassing many ENSO oscillations are required to understand the global picture of perennial tree crop production security. Combined with local context-specific studies on governance arrangements^16^, such approaches could be crucial for reducing future vulnerability of these industries to increasing volatility under anthropogenic climate change. The main barrier to this research is the availability of production data from state or commercial entities.

## Conclusions

Using cocoa production in Ghana as a model perennial tree crop system, we demonstrate that ENSO phase has a significant impact on crop production, likely mediated by simultaneous impacts on local climate. In a novel finding, we also show delayed effects of ENSO phase on production. Crucially, we demonstrate that the direction of production impacts has reversed over time, coinciding with changes in the climatic responses to ENSO, suggesting that anthropogenic climate change is altering how this perennial tree crop responds to climate shocks. We speculate that similar patterns are likely to occur in at least some other perennial tree crops, and urge for further research to emulate our straightforward “big data” approach in other crops to identify these patterns and contribute towards efforts to predict and manage the impacts of climate change on the millions of livelihoods dependent on this agricultural sector.

## Supplementary Information

Supplementary Information.

## Data Availability

Cocoa production data is available on request from the Ghana Cocoa Board, the corresponding author can facilitate this on request. All other data is freely available from the online databases cited.

## References

[1] Parry ML, Rosenzweig C, Iglesias A, Livermore M, Fischer G (2004). Effects of climate change on global food production under SRES emissions and socio-economic scenarios. Glob. Environ. Change.

[2] Rifai SW, Li S, Malhi Y (2019). Coupling of El Niño events and long-term warming leads to pervasive climate extremes in the terrestrial tropics. Environ. Res. Lett..

[3] Cai W (2014). Increasing frequency of extreme El Niño events due to greenhouse warming. Nat. Clim. Change.

[4] Wang B (2019). Historical change of El Niño properties sheds light on future changes of extreme El Niño. Proc. Natl. Acad. Sci..

[5] Malhi Y, Wright J (2004). Spatial patterns and recent trends in the climate of tropical rainforest regions. Philos. Trans. R. Soc. Lond. B. Biol. Sci..

[6] Surmaini E, Hadi TW, Subagyono K, Puspito NT (2015). Early detection of drought impact on rice paddies in Indonesia by means of Niño 3.4 index. Theor. Appl. Climatol..

[7] Jayakumar M, Rajavel M, Surendran U, Gopinath G, Ramamoorthy K (2017). Impact of climate variability on coffee yield in India—With a micro-level case study using long-term coffee yield data of humid tropical Kerala. Clim. Change.

[8] Gateau-Rey L, Tanner EVJ, Rapidel B, Marelli J-P, Royaert S (2018). Climate change could threaten cocoa production: Effects of 2015–16 El Nino-related drought on cocoa agroforests in Bahia, Brazil. PLoS ONE.

[9] Oettli P, Behera SK, Yamagata T (2018). Climate based predictability of oil palm tree yield in Malaysia. Sci. Rep..

[10] Li Y, Strapasson A, Rojas O (2020). Assessment of El Niño and La Niña impacts on China: Enhancing the early warning system on food and agriculture. Weather Clim. Extrem..

[11] Iizumi T (2014). Impacts of El Niño Southern Oscillation on the global yields of major crops. Nat. Commun..

[12] Food and Agriculture Organization of the United Nations. FAOSTAT statistical database [Rome]. (1997).

[13] Belsky JM, Siebert SF (2003). Cultivating cacao Implications of sun-grown cacao on local food security and environmental sustainability. Agric. Hum. Values.

[14] Potts, J. *et al. The state of sustainability initiatives review 2014: Standards and the Green Economy*. https://www.iisd.org/system/files/pdf/2014/ssi_2014.pdf (2014).

[15] Davey MK, Brookshaw A, Ineson S (2014). The probability of the impact of ENSO on precipitation and near-surface temperature. Clim. Risk Manag..

[16] Hirons, M. Understanding climate resilience in Ghanaian cocoa communities—Advancing a biocultural perspective. *J. Rural Stud.* 10 (2018).

[17] Yeh S-W (2018). ENSO atmospheric teleconnections and their response to greenhouse gas forcing. Rev. Geophys..

[18] Huang, B. *et al.* Extended Reconstructed Sea Surface Temperature, Version 5 (ERSSTv5): Upgrades, Validations, and Intercomparisons. *J. Climate* **30**(20), 8179–8205. 10.1175/JCLID-16-0836.1 (2017).

[19] Hersbach H (2020). The ERA5 global reanalysis. Q. J. R. Meteorol. Soc..

[20] Aragão, L. E. O. C. *et al.* Spatial patterns and fire response of recent Amazonian droughts. *Geophys. Res. Lett.* **34**, (2007).

[21] Hijmans, R. Boundary, Ghana, 2015. (2015).

[22] Water Research Institute, Ghana. Ghana—Country at a Glance: Water bodies. (1998).

[23] R Core Team. R: A Language and Environment for Statistical Computing. (2021).

[24] Pebesma E (2018). Simple features for R: Standardized support for spatial vector data. R J..

[25] Pebesma, E., Sumner, M., Racine, E., Fantini, A. & Blodgett, D. stars: Spatiotemporal Arrays, Raster and Vector Data Cubes. (2021).

[26] Hyndman RJ, Khandakar Y (2008). Automatic time series forecasting: The forecast package for R. J. Stat. Softw..

[27] Bates D, Mächler M, Bolker B, Walker S (2015). Fitting linear mixed-effects models using lme4. J. Stat. Softw..

[28] Bartoń, K. MuMIn: Multi-Model Inference. (2020).

[29] Lahive F, Hadley P, Daymond AJ (2018). The physiological responses of cacao to the environment and the implications for climate change resilience. A review. Agron. Sustain. Dev..

[30] Keil A, Zeller M, Wida A, Sanim B, Birner R (2008). What determines farmers’ resilience towards ENSO-related drought? An empirical assessment in Central Sulawesi, Indonesia. Clim. Change.

[31] Ruf F, Schroth G, Doffangui K (2015). Climate change, cocoa migrations and deforestation in West Africa: What does the past tell us about the future?. Sustain. Sci..

[32] Moser G (2010). Response of cocoa trees (Theobroma cacao) to a 13-month desiccation period in Sulawesi, Indonesia. Agrofor. Syst..

[33] Abahous H, Bouchaou L, Chehbouni A (2021). Global climate pattern impacts on long-term olive yields in Northwestern Africa: Case from Souss-Massa Region. Sustainability.

[34] Groeneveld JH, Tscharntke T, Moser G, Clough Y (2010). Experimental evidence for stronger cacao yield limitation by pollination than by plant resources. Perspect. Plant Ecol. Evol. Syst..

[35] Almeida Silva K, de Souza Rolim G, Borges Valeriano TT, da Silva Cabral de Moraes JR (2020). Influence of El Niño and La Niña on coffee yield in the main coffee-producing regions of Brazil. Theor. Appl. Climatol..

[36] Gutierrez L (2017). Impacts of El Niño-Southern Oscillation on the wheat market: A global dynamic analysis. PLoS ONE.

[37] Najafi E, Devineni N, Khanbilvardi RM, Kogan F (2018). Understanding the changes in global crop yields through changes in climate and technology. Earths Future.

