# Supplementary material for: Climate change alters impacts of extreme climate events on a tropical perennial tree crop
Source: Sci Rep. 2022 Nov 16;12:19653. doi: 10.1038/s41598-022-22967-7 (PMC9668817; doi:10.1038/s41598-022-22967-7)
Supplement: Supplementary file 1 — Supplementary Information. [file 41598_2022_22967_MOESM1_ESM.pdf]

Supplementary Material: Climate change alters impacts of extreme climate events on a tropical perennial tree crop

Thomas J. Creedy[a]*, Rebecca A. Asare[b], Alexandra C. Morel[c], Mark Hirons[d], John Mason[b], Yadvinder Malhi[d], Constance L. McDermott[d], Emmanuel Opoku[e], Ken Norris[a]
[a]Department of Life Sciences, Natural History Museum, London, UK
[b]Nature Conservation Research Centre, Accra, Ghana
[c]Department of Geography and Environmental Sciences, University of Dundee, UK
[d]Environmental Change Institute, School of Geography and the Environment, University of Oxford, UK
[e]Ghana Cocoa Board, Accra, Ghana
*Corresponding author: thomas@tjcreedy.co.uk +447843837201

Supplementary Methods

Climate data acquisition

ERA5 climate data was acquired from the Copernicus Climate Data Service using the CDS API in a custom python script. We acquired hourly data at 0.25° resolution between -3.5° to 1° longitude and 4.5° to 8.5° latitude, for the full period of the 1950 to 1978 preliminary back extension dataset, from 1979 to 2019 from the final release dataset, and 2019 to 2020 from the timely release dataset (accessed 2021-10-19) for the variables 2m temperature, total precipitation and evaporation as netcdf raster bricks. All variables were summarised by day for each grid cell, calculating the daily total for accumulating variables (precipitation and evaporation), and daily minimum, mean and maximum for temperature.

We defined Ghana's four climatological seasons as: minor wet, September and October; major dry, November - March; major wet, April - July; minor dry, August. All climate variables were then summarised over month and season, calculating total values for accumulating variables and minimum, mean and maximum for temperature. We calculated monthly Cumulative Water Deficit (CWD) for each ERA5 raster cell (Aragão et al 2007) based on monthly totals of precipitation and evaporation, resetting CWD to 0 for the wettest month for each cell or if rainfall exceeded twice the evaporation for a given month (Malhi et al., 2004a). Thus for each purchase year (Oct-Sep, see above) we generated 12 monthly and 4 seasonal values for each climatic metric. The minor wet season crosses the purchase year: we considered this as falling at the beginning of the purchase year rather than the end, as this has a more reasonable link to cocoa production. Finally, each climate metric was converted to anomalies by subtracting the mean value for the metric for a reference period, set to 1981-2010 to encompass only data from the final release ERA5 dataset. Mean values were computed across months and seasons to retain variation among months/seasons. The final dataset comprised climate data for the 70 cocoa purchase years 1950/51 to 2019/20.

Supplementary Results

Validation of the detrending approach

Supplementary Table 2 shows that for the vast majority of the individual time series within the district and regional datasets, the best fitting parameters for p (autoregression order), d (degree of differencing) and q (moving average) were equal to zero. While some individual time series do have non-zero values, this is to be expected by chance alone given the number of trials run, and the potential error generated by these data would only reduce the significance of downstream analyses.

For each dataset, a mixed effects model fitting production anomaly against the intercept with district or region as a random effect was determined to be singular with a tolerance of 0.0001, thus confirming that the detrending process removed sufficient variation between districts/regions.

ENSO-production relationships

We calculated cross-correlation between time series of detrended production and mamONI for the same purchase year and 12 delayed purchase years to identify potential instantaneous or delayed relationships between production and mamONI; these analyses show significant correlations at all years for the district dataset and several years including instantaneous for the regional dataset (Supplementary Figure 2).

ENSO-climate relationships

To examine climate impacts of ENSO with greater temporal resolution, we performed a cross-correlation on 3-monthly average ONI values against monthly temperature, precipitation and MWCD, including 36-month delays and leads. We observed that the climate of the purchase year prior to an El Niño is significantly cooler and wetter than average, with less drought, and vice-versa prior to a La Nina (Supplementary Figures 3 and 4).

Supplementary Figure 1 shows the results of multiple regressions fitting temperature, precipitation and (maximum) climatological water deficit (month: CWD, season: MCWD) against mamONI, with time period ("past" or "recent") as an interaction, for each month.

Supplementary Tables

Region	Data range	District	Data range
Ashanti	1947-2019	Agona	1999-2019

		Ampenim	1999-2019
		Antoakrom	1999-2019
		Bekwai	1999-2019
		Effiduase	1999-2019
		Juaso	1999-2019
		Konongo	1999-2019
		Mankranso	1999-2019
		New Edubiase	1999-2019
		Nkawie	1999-2019
		Nsokote	1999-2019
		Nyinahin	1999-2019
		Obuasi	1999-2019
		Offinso	1999-2019
		Tepa	1999-2019
Brong Ahafo	1960-2019 (excl. 1976)	Asumura	1999-2019
		Dormaa	1999-2019
		Goaso	1999-2019
		Hwidiem	1999-2019
		Kasapin	1999-2019
		Kukuom	1999-2019
		Nkrankwanta	1999-2019
		Sankore	1999-2019
		Sunyani	1999-2019
Central	1960-2019 (excl. 1976)	Agona Swedru	1999-2019
		Asikuma	1999-2019
		Assin Breku	1999-2019
		Assin Foso	1999-2019
		Cape Coast	1999-2019

			Nyinase	1999-2019
			Twifo Praso	1999-2019
Eastern	1947-2019		Achiase	1999-2019
			Akim Akoase	1999-2019
			Akim Oda	1999-2019
			Akim Ofoase	1999-2019
			Asamankese	1999-2019
			Kade	1999-2019
			Kibi	1999-2019
			Koforidua	1999-2019
			Nkawkaw	1999-2019
			Suhum	1999-2019
Volta	1947-2019		Hohoe	1999-2019
Western	1947-2019	Western North	Adabokrom	1999-2019
			Akontombra	1999-2019
			Asawinso	1999-2019
			Asempaneye	1999-2019
			Bodi	1999-2019
			Bonsu Nkwanta	1999-2019
			Debiso	1999-2019
			Essam	1999-2019
			Fosukrom	1999-2019
			Juabeso	1999-2019
			Sefwi Anhwiaso	1999-2019
			Sefwi Bekwai	1999-2019
			Sefwi Kaase	1999-2019
			Sefwi Wiawso	1999-2019
		Western South	Agona Amenfi	1999-2019

			Asankragwa	1999-2019
			Bogoso	1999-2019
			Dadieso	1999-2019
			Diaso	1999-2019
			Dunkwa	1999-2019
			Enchi	1999-2019
			Manso Amenfi	1999-2019
			Samreboi	1999-2019
			Takoradi	1999-2019
			Tarkwa	1999-2019
			Wassa Akropong	1999-2019

Supplementary Table 1: The 6 cocoa purchase regions and 68 cocoa purchase districts of Ghana, from which data was used in this study. The years of cocoa data available for use is shown; year denotes the start of the purchase year (i.e. 1976 = purchase year 1976/77). Note that at the regional level, the two Western regions are considered one, but at the district level, districts are split between Western North and Western South regions.

**Dataset**	**Parameter**	**Count of time series by parameter value**					
		**0**	**1**	**2**	**3**	**4**	**5**
**District** n=68	*p*	46	14	3*	3	2	0
	d	68*	0	0			
	q	65	0	3*	0	0	0
**Regional** n=6	*p*	4	1	1*	0	0	0
	d	6*	0	0			
	q	3	1	1*	1	0	0

*Supplementary Table 2: Results of ARIMA parameter search conducted on the time series of individual district or region production z-scores with mamONI as an external regressor. Counts record the number of individual district or region time series for each parameter and parameter value where that value resulted in the best ARIMA model under AICc. Counts of 0 denote a parameter value that was included in value searching but was not selected for any time series; blank cells (i.e. d >= 3) denote parameter values that were not tested. Cells with * denote starting values (i.e. p = 2, d = 0, q = 2). For all parameters in both datasets, 0 was the most frequent parameter value.*

Interaction	logLik	AICc	delta	weight
1986/87	-488.5993	999.9063	0	0.4074
1987/88	-488.8921	1000.4919	0.5856	0.3040
1985/86	-490.1034	1002.9145	3.0082	0.0905
1984/85	-490.2422	1003.1922	3.2859	0.0788
cont. year	-490.7982	1004.3041	4.3978	0.0452
1983/84	-491.0935	1004.8948	4.9886	0.0336
1989/90	-491.9460	1006.5994	6.6934	0.0143
1990/91	-492.4793	1007.6664	7.7602	0.0084
1994/95	-493.0507	1008.8092	8.9030	0.0048
1993/94	-493.1539	1009.0155	9.1092	0.0043

*Supplementary Table 3: Model selection to identify most parsimonious piecewise multiple regression for explaining change in production response to mamONI over time in the regional dataset. Showing top 10 models (out of 74) only. 73 models took the form pa ~ mamONI0 * pp[year] + mamONI0 * pp[year] + mamONI0 * pp[year] + mamONI0 * pp[year], where pa = production anomaly, $mamONI_t$ = mamONI at year t, and pp[year] = a categorical variable dividing the data piecewise into (i) years up to and including purchase year [year] and (ii) purchase years following [year]. 1 model instead fitted year as a continuous variable, i.e. pa ~ mamONI0 * year + mamONI0 *year + mamONI0 * year + mamONI0 * year. All models had d.f. = 11 in the model selection, total n = 412 in initial model fitting. The* **Interaction** *column gives the value of the piecewise categorical variable, i.e. [year] in pp[year], or notes the exceptional model with year fit as a continuous variable;* **logLik** *gives the log-likelihood and* **AICc** *the corrected Akaike Information Criterion of the corresponding model;* **delta** *the change in AICc from the top model and* **weight** *the relative Akaike weight of each model.*

Supplementary Figures

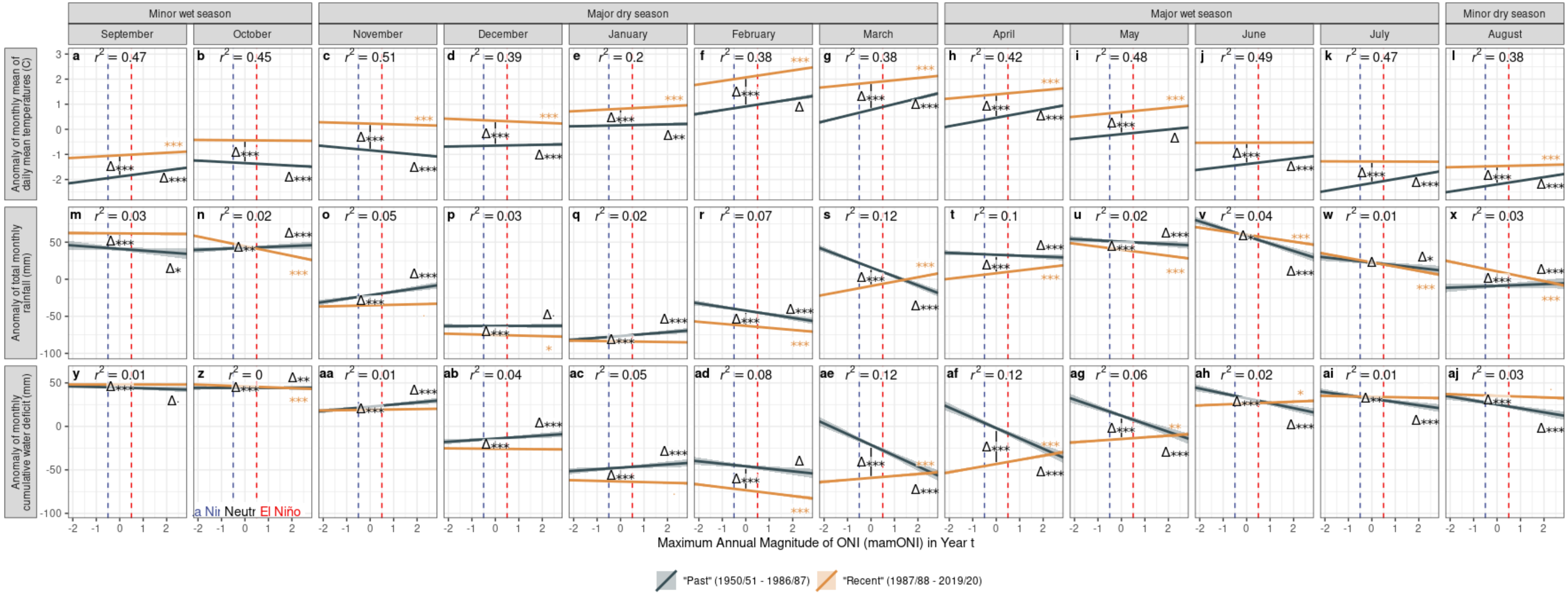

*Supplementary Figure 1: The response of climate to mamONI in different months during the purchase year, grouped into two sets of years corresponding to the best fitting break-point in the production data. Vertical dashed lines delineate La Nina (mamONI <= -0.5), Neutral (-0.5 < mamONI < 0.5) and El Nino (mamONI >= 0.5) conditions. Lines show the best linear fits and standard errors derived from 36 individual regressions of monthly climate against mamONI, with an interaction term fitting the year category. Significance stars denote p-values derived from these models (***: p<0.001, **: p < 0.01, *: p < 0.05, .: p < 0.1): (i) difference in means between year groups (delta in centre of plot), (ii) difference of the 1987/88-2018/19 slope from 0 (orange stars), (iii) difference between slopes (delta at right of plot). Adjusted R squared values are displayed.*

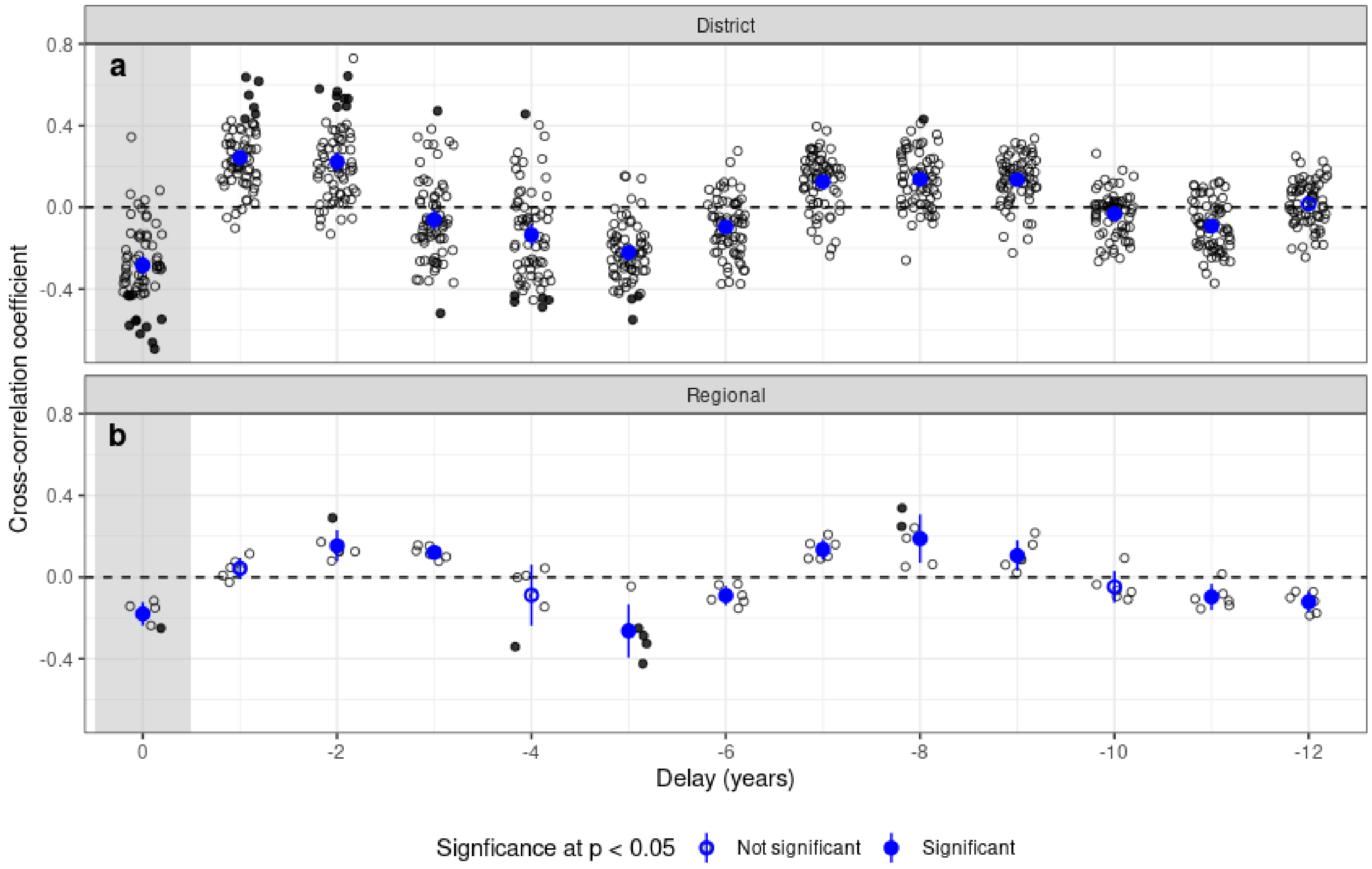

Supplementary Figure 2: Cross-correlation of production z-scores with instantaneous and delayed annual values of mamONI to illustrate potential delays in the response of cocoa production to El Nino from instantaneous (Delay = 0, shaded) to 12 (i.e. a response in production to ONI twelve years prior). Cross-correlations were performed on the time series of production z-scores for each district (a, top panel) or region (b, bottom panel) separately (black points, filled if correlation is significant at $p < 0.05$). Significant positive values indicate an increase in production with El Nino and decrease with La Nina, compared with the average trend of production. Blue points and error bars show the mean and 95% interval for each delay, filled blue points indicate that the mean is significantly different from zero (t test, $p < 0.05$). Horizontal jitter added to black points to avoid overfitting. Horizontal dashed line denotes cross-correlation coefficient of 0.

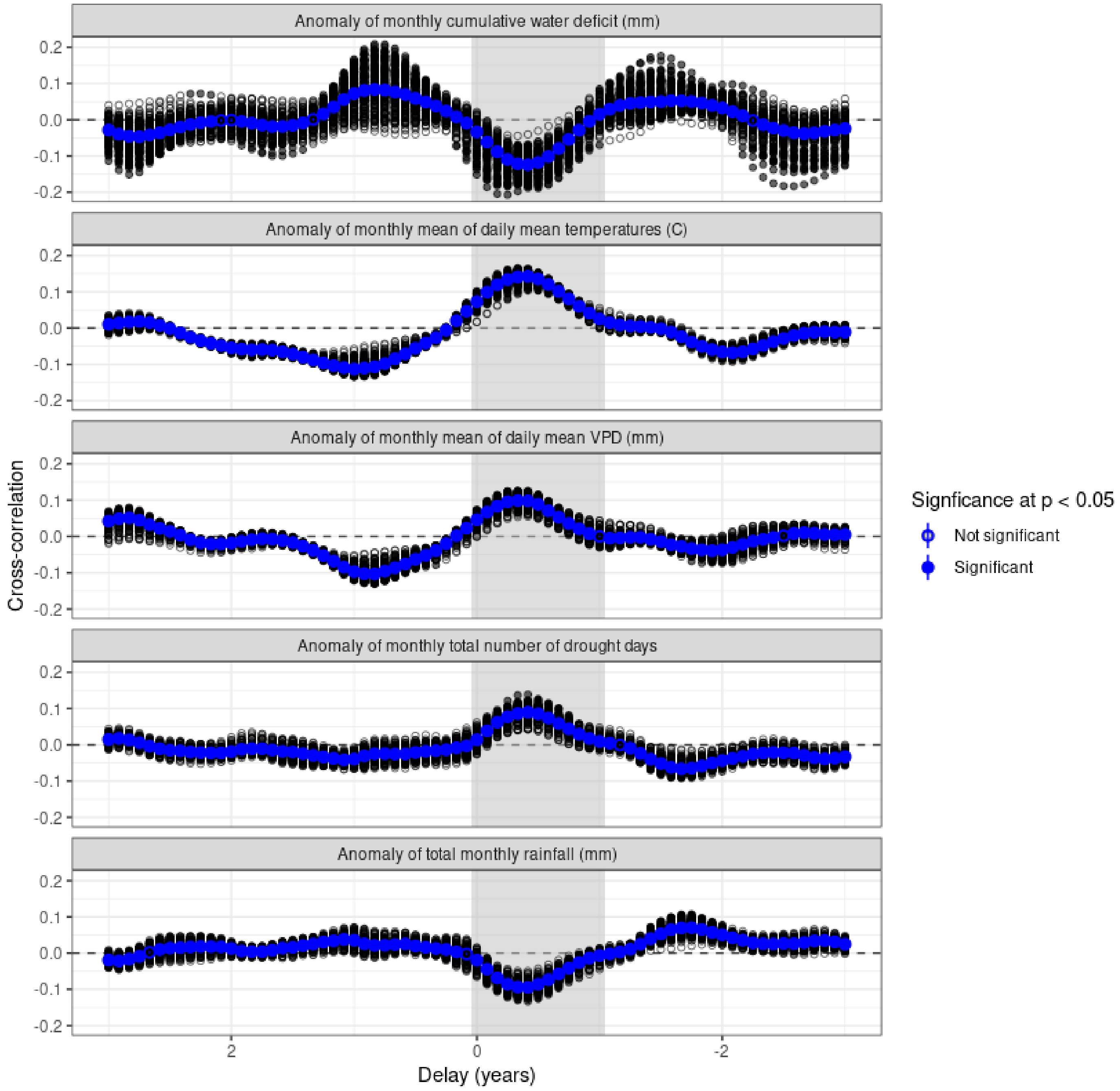

Supplementary Figure 3: Cross-correlation of monthly climate anomalies with leading/delayed monthly values of ONI to illustrate potential delays in the response of climate to ENSO from +36 to -36 months (i.e. respectively, leading responses of 36 months prior to ENSO, positive values on x axis, and delayed responses of 36 months after ENSO, negative values on x axis). Note that the x axis is reversed for consistency of treatment of time (left = leading, right = delayed) across all plots. Cross-correlations were performed on the time series of climate anomalies for each grid square separately (black points, filled if correlation is significant at p < 0.05). Significant positive values indicate an increase in that climate variable with El Nino and decrease with La Nina, compared with the average trend. Blue points and error bars show the mean and 95% interval for each lag, filled blue points indicate that the mean is significantly different from zero (t test, p < 0.05). Horizontal jitter added to black points to avoid overfitting. Horizontal dashed line denotes cross-correlation coefficient of 0. Shaded region denotes the 12 months of the buying year over which mamONI is calculated and cocoa production data is aggregated.

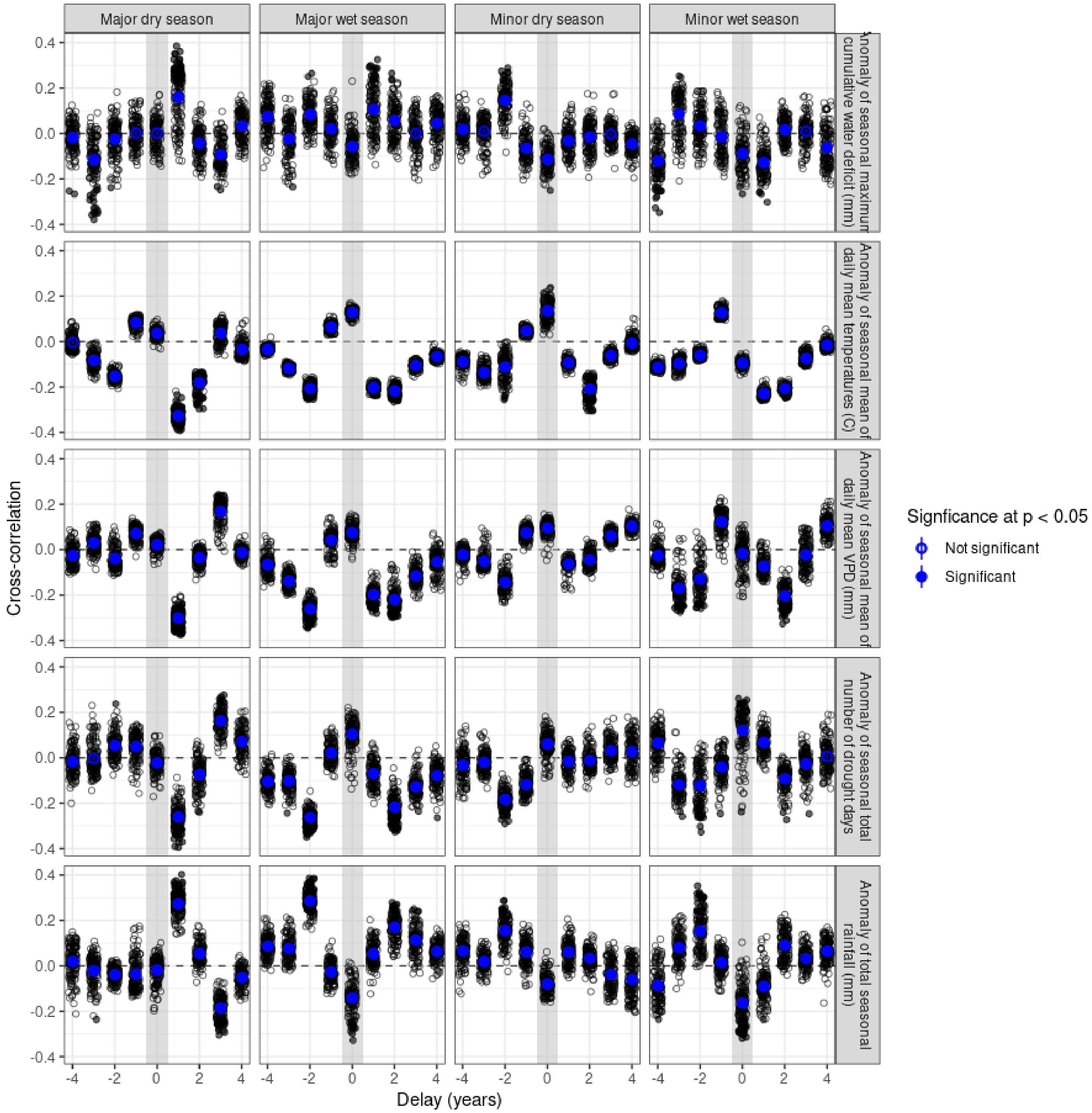

Supplementary Figure 4: Cross-correlation of annual climate anomalies with lagged and leading values of mamONI to illustrate potential lags in the response of climate to ENSO from +4 to -4 years (i.e. respectively, leading responses of 4 years prior to ENSO, positive values on x axis, and delayed responses of 4 years after ENSO, negative values on x axis). Note that the x axis is reversed for consistency of treatment of time (left = leading, right = delayed) across all plots. Cross-correlations were performed on the time series of climate anomalies for each grid square separately (black points, filled if correlation is significant at $p < 0.05$). Significant positive values indicate an increase in that climate variable with El Nino and decrease with La Nina, compared with the average trend. Blue points and error bars show the mean and 95% interval for each lag, filled blue points indicate that the mean is significantly different from zero (t test, $p < 0.05$). Horizontal jitter added to black points to avoid overfitting. Horizontal dashed line denotes cross-correlation coefficient of 0.
